# Serum vascular endothelial growth factor is a biomolecular biomarker of severity of diabetic retinopathy

**DOI:** 10.1186/s40942-019-0179-6

**Published:** 2019-10-01

**Authors:** Sukriti Ahuja, Sandeep Saxena, Levent Akduman, Carsten H. Meyer, Peter Kruzliak, Vinay K. Khanna

**Affiliations:** 1 Department of Ophthalmology, King George’s Medical University, Lucknow, U.P. 226003 India; 2 Department of Ophthalmology, Saint Louis University, St. Louis, USA; 3 Department of Ophthalmology, Pallas Klinik, Aarau, Switzerland; 4 Development of Cardiovascular Diseases, International Clinical Research Center, St. Anne’s University Hospital and Masaryk University, Brno, Czech Republic; 5 Developmental Toxicology Division, CSIR-Indian Institute of Toxicology Research, Lucknow, India

**Keywords:** Vascular endothelial growth factor, Diabetic retinopathy, Severity, Biomarker, Receiver operator characteristic curve, Area under curve

## Abstract

**Background:**

Elevated serum vascular endothelial growth factor (VEGF) levels are associated with diabetic retinopathy (DR). Serum VEGF levels correlate with vitreous levels. Neuroretinal changes occur even before the appearance of vascular signs in DR. Role of VEGF as a biomarker for DR has not been assessed. Serum VEGF as a biomarker for severity of DR, was evaluated for the first time.

**Methods:**

Consecutive cases of type 2 diabetes mellitus [without DR, (no DR, n = 38); non-proliferative DR, (NPDR, n = 38); proliferative DR, (PDR, n = 40)] and healthy controls (n = 40) were included. Serum VEGF was measured using enzyme linked immunosorbent assay. Accuracy of VEGF as a biomarker for severity of retinopathy was measured using the area under the receiver operator characteristic (ROC) curve.

**Results:**

Serum VEGF levels in controls, No DR, NPDR and PDR groups showed significant incremental trend from 138.96 ± 63.37 pg/ml (controls) to 457.18 ± 165.69 pg/ml (PDR) (F = 48.47; *p* < 0.001). Serum VEGF levels were observed to be significantly elevated even before DR had set in clinically. ROC for serum VEGF levels was significant in discriminating between the cases and the controls and had good accuracy in discerning between subjects with and without retinopathy. The area under curve (AUC ± SE) for discrimination was significant: (a) cases and controls (n = 156): AUC = 0.858 ± 0.029, *p* < 0.001; (b) DR (NPDR + PDR) and No DR (n = 116): AUC = 0.791 ± 0.044, *p* < 0.001; and (c) NPDR and PDR (n = 78): AUC = 0.761 ± 0.056, *p* < 0.001, with over 90% projected sensitivity and specificity at various cut off values.

**Conclusion:**

Serum VEGF level is a simple, effective laboratory investigative test in predicting the onset of DR in eyes showing no evidence of DR and serves as a reliable biomolecular biomarker for severity of DR.

## Background

Diabetic retinopathy (DR) is a sight-threatening microvascular complication of diabetes mellitus [1]. It is the leading cause of preventable blindness in working-age adults. The potential risk of blindness in an individual with diabetes is 2.4 times higher than that of an individual without diabetes [2, 3]. The prevalence of DR directly correlates with the duration of diabetes, ranging from 28.8% in people with diabetes for < 5 years to 77.8% in people with more than 15 years of diabetes [4].

Vascular endothelial growth factor (VEGF) is a part of a subfamily of growth factors, functioning as signalling proteins, and involved in angiogenesis. VEGF serves as a biomolecule and is secreted from retinal pigment epithelial cells, pericytes, astrocytes, muller cells, glial cells and endothelial cells [5]. VEGF induces retinal intercellular adhesion molecule-1 (ICAM-1) expression and retinal leucocyte adhesion, leading to blood retinal barrier (BRB) breakdown, capillary non-perfusion and endothelial cell injury [6]. The balance between VEGF and angiogenic inhibitors is the chief determinant of angiogenesis and proliferation in DR [7]. Within the kidney, VEGF is predominantly expressed by podocytes [8]. Increased serum VEGF levels stimulate apoptosis-promoting reactive oxygen species (ROS) generation and causes endothelial cell activation. Thus, adversely affecting the endothelial cells [9, 10].

Published literature shows that VEGF correlates significantly with DR [11–20]. Our earlier work highlighted that serum VEGF levels correlated with severity of DR, increase in macular thickness and retinal photoreceptor ellipsoid zone disruption [21]. The present study was undertaken to evaluate serum VEGF as a biomolecular biomarker for severity of DR, for the first time.

## Materials and methods

The authors confirm adherence to the tenets of the Declaration of Helsinki. An institutional review board clearance was obtained. A written informed voluntary consent was obtained from all the study subjects. Tertiary care-centre based case control study included consecutive cases with type 2 diabetes mellitus and healthy controls. Diabetes was diagnosed according to American diabetes association criteria as a fasting plasma glucose level ≥ 126 mg/dL (7.0 mmol/L) or 2-h post prandial glucose level ≥ 200 mg/dL (11.1 mmol/L) during an oral glucose tolerance test [22].

Information regarding age, gender, and disease duration was recorded. Study subjects with any of the following conditions were excluded: ocular or systemic diseases which might affect the retinal vascular pathology, any previous intravitreal injection(s), surgical or laser interventions, and systemic diseases that affect VEGF levels such as inflammatory disorders (e.g., asthma and rheumatoid arthritis), ischemic heart disease, current or planned dialysis and malignancies.

The best-corrected visual acuity was recorded on Snellen’s visual acuity chart and logMAR scale. All the study subjects underwent detailed fundus evaluation using stereoscopic slit-lamp biomicroscopy and indirect ophthalmoscopy. Digital fundus photography and fluorescein angiography were done using a Zeiss fundus camera FF 450 Plus (Carl Zeiss Meditech AG, Jena, Germany). Based on the fundus photography and fluorescein angiography, cases were divided into three groups: diabetes mellitus patients without retinopathy (No DR; n = 38), with non-proliferative diabetic retinopathy (NPDR; n = 38), and with proliferative diabetic retinopathy (PDR; n = 40) according to the early treatment of diabetic retinopathy study (ETDRS) classification [23]. Healthy controls, of similar age (n = 40), with no diabetes mellitus were also included. Power of the study was 80%.

Seven ml blood sample was collected from the study subjects. The glass tubes containing blood were left on a stand for 30 min to allow the blood to clot. Subsequently, the samples were centrifuged at 1000×g for 10 min, and the serum was separated into other tubes.

The samples were kept at − 80 °C till assay of VEGF was done. Human VEGF enzyme linked immunosorbent assay (ELISA) kit procured from Invitrogen (Carlsbad, CA) was used to perform VEGF assay. A standard protocol provided with the kit was used to prepare the reagents in the kit. An incubation buffer (50 µl) was then added to the multiwell plates precoated with a monoclonal antibody specific for the VEFG protein.

The VEGF standard provided with the kit was reconstituted with the standard diluent buffer. Sequential dilutions of the VEGF standard (0, 23.4, 46.9, 93.8, 188,375, 750, 1500 pg/ml) were done according to the instructions and run in parallel. The regular amount (100 µl) was added to the appropriate microtiter wells. Diluent buffer (50 µl) and a serum sample (50 µl) were further added to the well. Incubation of the plate was done at room temperature for 2 h.

The plate contents were removed using multichannel pipettes and the plate was washed with the washing buffer four times to remove any unbound antigens (proteins). Biotinylated *Hu VEGF* (Biotin Conjugate, 100 µl) was added to each well. The plate was again incubated for 1 h at room temperature and the contents were removed from the plate, which was soon after washed four times.

A 100 µl substrate of a streptavidin HRP working solution was then added to each well, and the plate was incubated for 30 min at room temperature. The contents were removed from the plate, which was once more washed four times. A chromogen solution (100 µl) was subsequently added to each well to stabilize the chromogen that turned blue. Following this, incubation of the plate was done for 30 min at room temperature in the dark. 100 µl of the solution provided in the kit was added to each well to stop the reaction. The blue colour that developed earlier turned yellow and the intensity of the colour was read with an ELISA plate reader (Synergy HT, Biotech, Winooski, VT) at 450 nm.

The calibration curve of the standard VEGF was plotted against the VEGF with absorbance on the x-axis and concentration on the y-axis. The VEGF concentration in the serum sample was calculated based on the standard curve. The values were expressed as pg/ml.

Statistical analysis: Data was analysed using Statistical Package for Social Sciences (SPSS) version 21.0. Chi square test and ANOVA followed by Tukey’s HSD test was used for univariate intergroup comparisons. Discriminant value of VEGF was evaluated using receiver operator characteristic (ROC) curve analysis. Linear regression was performed for multivariate analysis. A ‘*p*’ value less than 0.05 was considered statistically significant. 

## Results

Table 1 shows the characteristics of the study groups. There was no significant difference with respect to demographic features, including age and gender, between the cases and controls (*p* > 0.05). According to ETDRS classification, the cases with retinopathy (n = 78) were classified as mild NPDR (n = 7), moderate NPDR (n = 19), severe NPDR (n = 12), early PDR (n = 16) and advanced PDR (n = 24). Newman–Keuls test showed that mean central subfield thickness (CST) was significantly different among the study groups (*p* < 0.001).

**Table 1:** Characteristics of study groups

S. no	Characteristic	Controls (n = 40)	No DR (n = 38)	NPDR (n = 38)	PDR (n = 40)
1.	Age (years) (mean ± SD)	52.95 ± 7.49	52.11 ± 5.84	55.21 ± 4.78	53.58 ± 6.87
2.	Gender				
	Male	26	24	26	25
	Female	14	14	12	15
3.	Duration of diabetes in years (mean ± SD)	0	7.16 ± 6.04	10.26 ± 5.82	11.08 ± 4.55
4.	Glycated Hb (%) (mean ± SD)	5.35 ± 0.1	7.42 ± 0.19	8.48 ± 0.28	8.90 ± 0.18
5.	Central subfield thickness (mean ± SE)	247.9 ± 2.6	251.7 ± 4.3	304.7 ± 22.5	455.9 ± 19.36
6.	S. urea (mg/dl)	33.26 ± 0.8	38.03 ± 2.1	37.96 ± 0.9	39.89 ± 1.1
7.	S. creatinine (mg/dl)	0.96 ± 0.01	1.12 ± 0.02	1.11 ± 0.02	1.61 ± 0.01

**Fig. 1:**
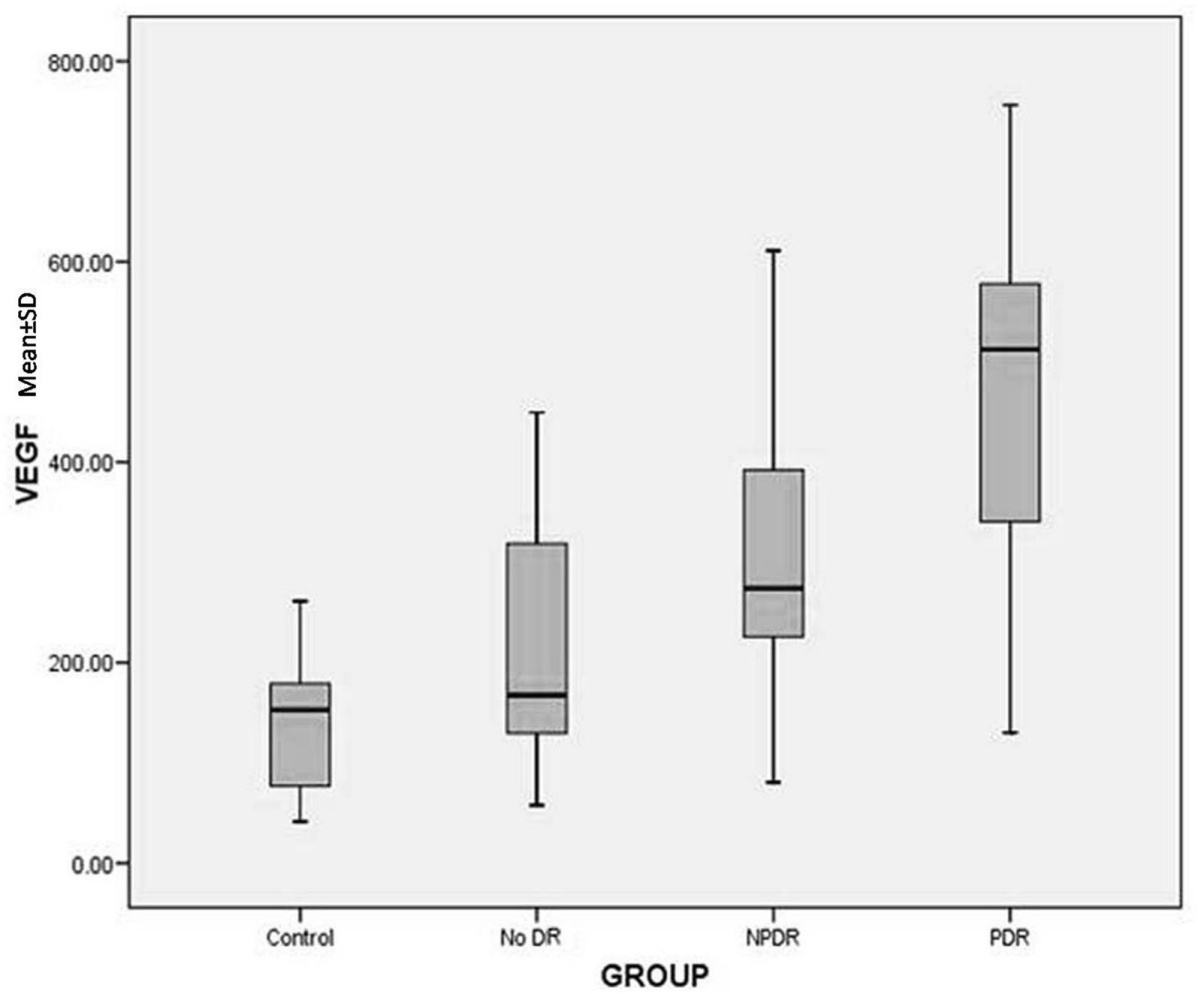
Box plot showing mean serum VEGF ± SD in controls (n = 40), no diabetic retinopathy (no DR, n = 38), non-proliferative diabetic retinopathy (NPDR, n = 38) and proliferative diabetic retinopathy (PDR, n = 40)

Serum VEGF levels in controls, No DR, NPDR and PDR groups showed significant incremental trend (F = 48.474; *p* < 0.001). The mean serum VEGF Levels in mild NPDR (189.48 ± 35.37), moderate NPDR (277.29 ± 56,67), severe NPDR (434.34 ± 66.67) also showed an incremental trend. Using Tukey’s HSD test, difference between all the groups were found to be significant (*p* < 0.05) (Fig. 1).

**Fig. 2:**
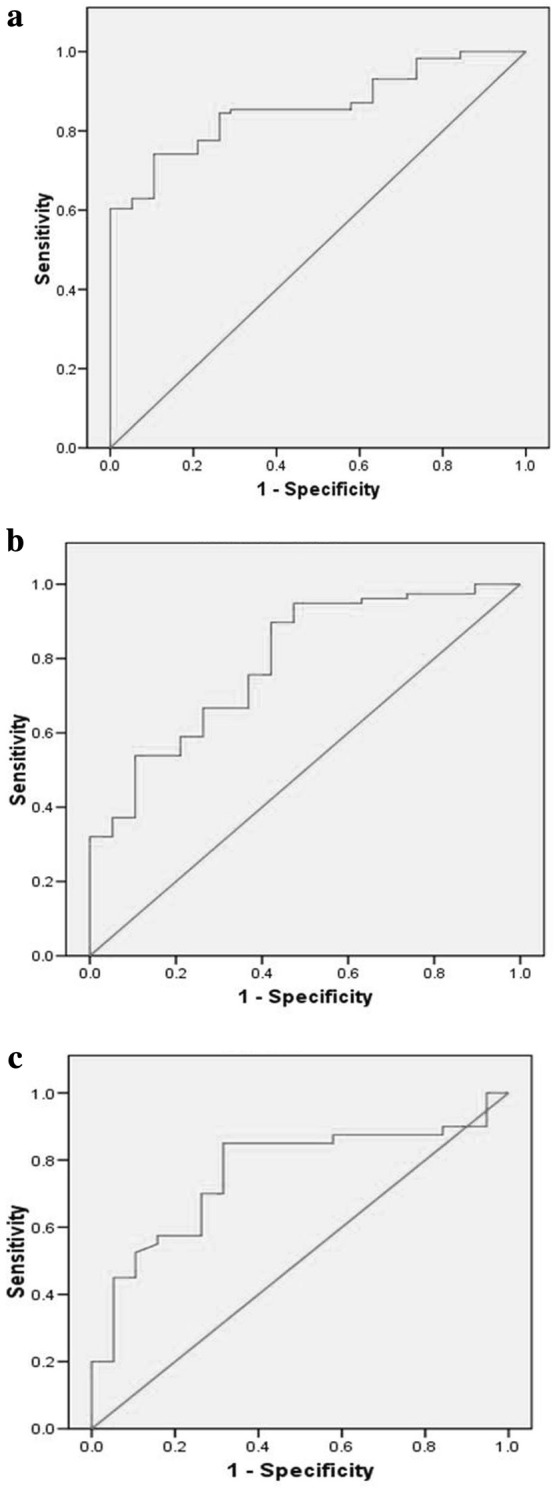
**a** ROC curve for distinction between cases and controls, AUC = 0.858 (*p* < 0.001). **b** ROC curve for distinction of diabetic retinopathy and no retinopathy in cases, AUC = 0.791 (*p* < 0.001). **c** ROC curve for distinction between non-proliferative and proliferative diabetic retinopathy, AUC = 0.761 (*p* < 0.001)

For VEGF, ROC curve projections were performed to evaluate, the area under curve values (AUC ± SE) for discrimination between (a) cases (No DR + NPDR + PDR) and controls (n = 156): AUC = 0.858 ± 0.029, *p* < 0.001, projected high sensitivity = 91.4% (cut-off VEGF value = 124.05), projected high specificity = 94.7% (cut-off VEGF value = 253.08); (b) DR (NPDR + PDR) and No DR (n = 116): AUC = 0.791 ± 0.044, *p* < 0.001, projected high sensitivity = 92.3% (cut-off VEGF value = 177. 64), projected high specificity = 94.7% (cut-off VEGF value = 418.39); and (c) NPDR and PDR (n = 78): AUC = 0.761 ± 0.056, *p* < 0.001, projected high sensitivity = 90.3% (cut-off VEGF value = 177.64), projected high specificity = 94.7% (cut-off VEGF value = 533.0) (Fig. 2a–c).

Multivariate regression analysis was performed to study the association of VEGF with independent variables namely, study groups, age, sex, and duration of diabetes mellitus. A significant association was observed between the study groups with VEGF levels (*p* < 0.001). The association of VEGF levels and age (*p* = 0.109), sex (*p* = 0.291), and duration of diabetes mellitus (*p* = 0.798) was not significant.

## Discussion

Our study evaluated the status of serum VEGF levels in cases with type 2 diabetes mellitus and its association with the presence and severity of the retinopathy. VEGF is an important regulator of ocular angiogenesis and vascular permeability and is found to be involved in the pathogenesis of several complications of DR such as DME and PDR [5].

The permeability of retinal capillaries is altered by VEGF as it increases the phosphorylation of proteins involved with tight junctions, such as zonula occludens [24]. Our earlier study showed that increased levels of serum VEGF correlated with occurrence of DME and increased central subfield thickness and cube average thickness on spectral-domain optical coherence tomography [21].

VEGF regulates developmental and pathological angiogenesis [24, 25]. It activates and stimulates the endothelial cells, which results in degradation of basement membrane. Endothelial cell migration occurs, which is followed by synthesis of basement membranes for the newly formed capillaries [26]. Our earlier study showed that significantly increased levels of ICAM-1 correlated with severity of DR [21]. Increased levels of ICAM-1 cause vascular endothelial damage with formation of acellular capillaries, which further leads to retinal ischemia and up regulation of VEGF. High levels of VEGF lead to retinal neovascularization and PDR [14]. The duration and amount of VEGF exposure required for BRB breakdown is less than that required for neovascularization [27]. Elevated levels of ICAM-1 and VEGF come into play even before the signs of PDR have set in. The damage caused by them correlates with the duration of the disease. Increase in the expression of ICAM-1 caused by VEGF has been studied using animal models [6, 28–30]. Serum VEGF levels have also been shown to correlate with the glycemic control of the patients [31].

In the present study, mean serum VEGF values in controls, No DR, NPDR and PDR groups showed significant incremental trend from 138.96 ± 63.37 pg/ml (controls) to 457.18 ± 165.69 pg/ml (PDR) signifying that serum VEGF levels increased as severity of DR increased. A previous study had concluded that there was a reduction in the thickness of ganglion cell layer and retinal nerve fiber layer in diabetic patients without diabetic retinopathy changes. The study suggested that neuroretinal changes occur before the appearance of vascular signs in diabetic retinopathy [32]. Our study highlights that significantly elevated serum VEGF levels come into play even before the clinical signs of DR appear. This novel finding facilitates a better understanding of the disease process and helps in predicting the onset of DR.

In the present study, multivariate analysis showed that age, sex and duration of diabetes mellitus had no confounding effect on VEGF expression. Significant association was observed between VEGF levels and severity of retinopathy. Accuracy of VEGF as a biomarker for severity of retinopathy was measured using the area under the ROC curve. ROC curve evaluated the diagnostic value of serum VEGF for use as a novel biomolecular biomarker for severity of DR. ROC for serum VEGF levels is significant in discriminating between the cases and the controls and has good accuracy in discerning between subjects with and without retinopathy. The AUC values for discrimination between various study groups showed serum VEGF levels to be reliable and sensitive biomarker of severity of DR, with over 90% projected sensitivity and specificity at various cut off values.

It was highlighted in an earlier study that the mean vitreous and serum VEGF levels were significantly higher in cases of NPDR and PDR as compared to controls. A significant correlation between vitreous and serum VEGF levels was also observed, though it is not a perfect numerical correspondence, when each organ of a diseased body is assessed in regard to the VEGF levels [33, 34]. Hence, estimation of serum VEGF, with AUC cut-off levels, help in evaluating the occurrence/progression of DR to the next level.

In a clinical setting, estimation of serum VEGF levels may be used as an effective laboratory test in predicting the onset of DR in eyes showing no evidence of DR.

## Conclusion

Serum VEGF levels serve as a simple, reliable, physician-friendly, and easy to comprehend biomolecular biomarker for severity of DR. Significantly elevated levels of VEGF come into play even before the evidence of DR. Estimation of serum VEGF is a useful laboratory test for predicting the onset of DR. The only limitation of the study was small sample size.

## Abbreviations

VEGF: vascular endothelial growth factor
DR: diabetic retinopathy
DME: diabetic macular oedema
NPDR: non-proliferative diabetic retinopathy
PDR: proliferative diabetic retinopathy
